# Nanomaterials in the Wound Healing Process: New Insights and Advancements

**DOI:** 10.3390/pharmaceutics16030300

**Published:** 2024-02-21

**Authors:** Tanikan Sangnim, Vivek Puri, Divya Dheer, D. Nagasamy Venkatesh, Kampanart Huanbutta, Ameya Sharma

**Affiliations:** 1 Faculty of Pharmaceutical Sciences, Burapha University, 169, Seansook, Muang, Chonburi 20131, Thailand; tanikan@go.buu.ac.th; 2 Chitkara University School of Pharmacy, Chitkara University, Baddi 174103, Himachal Pradesh, India; divya.dheer@chitkarauniversity.edu.in; 3 Chemical Biology Unit, Institute of Nano Science and Technology, Knowledge City, Sector 81, Mohali 140306, Punjab, India; 4 JSS College of Pharmacy, JSS Academy of Higher Education & Research, Ooty 643001, Tamil Nadu, India; nagasamyvenkatesh@jssuni.edu.in; 5 Department of Manufacturing Pharmacy, College of Pharmacy, Rangsit University, Pathum Thani 12000, Thailand; kampanart.h@rsu.ac.th

**Keywords:** nanomaterials, wound healing, wound management, nanoparticles, antimicrobial

## Abstract

Wounds, which are becoming more common as a result of traumas, surgery, burns, and chronic illnesses like diabetes, remain a critical medical problem. Infectious bacteria impact the healing process, particularly if its biofilm (biological films) leads to a prolonged effect. Nanomaterials have emerged as promising candidates in the field of wound healing due to their unique properties and versatile applications. New insights into the interactions between nanomaterials and wound microenvironments have shed light on the mechanisms underlying their therapeutic effects. However, a significantly minimal amount of research has been carried out to see if these nanomaterials significantly promote the wound healing process. In this review, we provided an outline of the various types of nanomaterials that have been studied for healing wounds and infection prevention. Overall, the utilization of nanomaterials in wound healing holds great promise and continues to evolve, providing new opportunities for the development of effective and efficient wound care therapies.

## 1. Introduction

The skin is the body’s primary organ, having a multitude of activities that tend to range from the immunologic and sensory to insulating and regulating moisture [1]. Because of the documented frequency of accidents, surgeries, burns, and chronic illnesses like diabetes, the prevalence of various injuries remains a critical problem [2]. According to the World Health Organization’s (WHO) most recent data, burn wounds alone cause more than 300,000 fatalities per year. After skin injury, the skin’s immune and defensive processes are compromised; as a result, germs can readily infect the wound site, resulting in chronic infection as well as septic shock unless the invaders spread throughout the body [3]. Furthermore, pathogenic microbes can induce healing delays by forming a biofilm, which can lead to treatment problems owing to resistance to antibiotics and life-threatening consequences [4]. Simultaneously, due to limited antibiotic treatment choices, the emergence of multidrug-resistant (MDR) pathogens has posed a serious therapeutic issue [5]. For a wound, the longer the duration that it takes to heal, the greater the chance of a substantial adverse occurrence, such as amputation, organ loss, or death [6].

Antimicrobial-resistance (AMR) and multidrug-resistance (MDR) illnesses will cause more deaths than all forms of cancer combined, as according to the Centers for Disease Control and Prevention (CDCP). AMR is predicted to cause more than 10 million deaths per year by 2050 [7,8]. Traditional antibacterial medications are being used to treat wound infections, which pose a number of clinical problems. As a result, it is vital to create innovative dressing materials that do not rely on current antibiotics [8].

**Figure 1:**
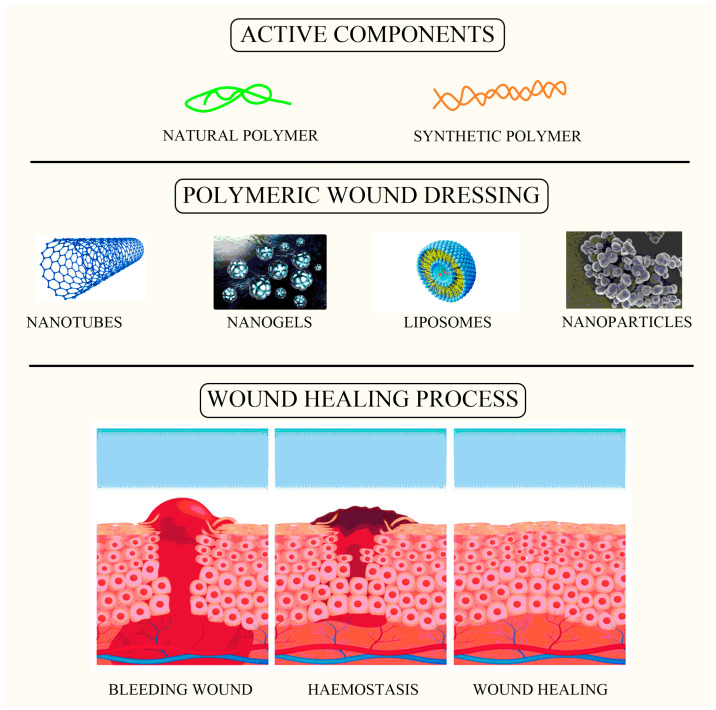
Image showing active components loaded within wound dressing as a carrier for the wound healing process.

Over the last two decades, the use of nanomaterials (NMs), specifically novel formulations such as nanoparticles formulated for wound healing processes and infection management, has expanded dramatically [9]. The scaffolding of nanoparticles for wound healing has recently gained a lot of attention as a targeted approach to advanced dressings [10]. Antimicrobial NMs currently come in a range of forms, including polymers such as lipids in liposomes, cellulose, and mesoporous silica, which serve as carriers for antimicrobial and wound healing agents (Figure 1) [11]. Metal/metalloid-based nanoparticles (MNPs) are another type of antibacterial NM that has been shown to help with wound healing in various studies [12]. Their great antibacterial efficacy could indeed, or might not, be intimately correlated to wound healing. Although the concept of nano-antibiotics (nanobots) has been synthesized for at least a decade, and there are several recent evaluations of nanomaterial utilization as antimicrobials or antiseptics available, there has been less focus on whether they truly enhance wound healing results [13]. Although there are several evaluations on the antibacterial effectiveness of different nanomaterials, only a few articles on nanomaterials specifically utilized to enhance wound healing have been published [14].

## 2. Wound Processes

To illustrate how nanomaterials could impact healing in context, here is a brief overview of wound subtypes and the healing process [15]. Disruption to the viability of underlying biologic and organic tissues, including skin and mucous membranes, is characterized as a wound. Burns, incisions, lacerations, contusions, abrasions, and combinations of these forms of trauma are all conceivable, and the maintenance of wound dressings for each type is vital to prevent infection and additional damage [16]. Wound categorization is carried out using a variety of approaches depending on cleanliness, wound state, and healing period [17]. Based on wound cleanliness and infection level, the CDC divides wounds into four categories. (1) Clean wounds: closed and free of infection and inflammation. (2) Cleanly contaminated or uncommonly contaminated; there is no remarkable contamination. (3) Infected: fresh, open, and also without exudate inflammation. (4) Highly infected wounds: the most frequent microorganisms are found in these wounds, which often originate from bad care of traumatic wounds [18,19,20].

Wounds are further divided into two groups: those that are infected and those that are not.

- Acute wounds: short-term (more prevalent), and sometimes inflicted by radiation, shock (electrical), excessive, or mechanical injury.
- Chronic wounds: long-term, and frequently a consequence of chronic disorders like diabetes [21,22].

## 3. Wound Healing Process

Healing is multifaceted, and it can go forward as well as backward depending on a variety of factors, including the patient’s external and internal surroundings/conditions [23]. Wound healing is split into four stages that overlap [24].

### 3.1. Hemostasis

This step occurs quickly, in order to create a blood clot and block blood flow [25].

### 3.2. Inflammation

Inflammation is managed during this phase, and organisms and cell debris are evacuated from the affected area. When activated by thrombin, platelets release a slew of growth factors including signals for storing white blood cells, nutrients, and more growth factors that further promote rapid wound and skin healing as well as strengthen immunity against infection [26].

### 3.3. Proliferation

Beginning with proangiogenic chemicals generated by inflammatory blood platelets, this phase repairs the wound. After that, angiogenesis takes place, fibroblasts multiply, and elastin is formed. By holding the wound edge, fibroblasts develop into myofibroblasts, forcing the wound region to constrict [27].

### 3.4. Maturation

With the aid of collagen fibers, the wound becomes completely closed. The debris and damaged cells that were used to heal the wound are cleared away by a process called apoptosis and thus collagen cross-linking strengthens the skin of the wounded region [28].

Hemostasis, inflammation, proliferation, and remodeling are the four phases of the complex wound healing process [29,30]. The therapeutic impact, dose, depth, and various approaches are the key factors for rational. Scientists have been investigating technologies that can both maintain a wet environment and kill bacteria [31]. There are several different engineered wound healing routes using a wide range of natural and synthetic polymeric materials and their amalgamation [32]. 

Here too, natural polymers with important biological roles have found their way into a wide variety of wound healing approaches [33]. For its ability to maintain a moist, protective environment for the tissues to heal in, collagen has been heralded as a superior, mechanically strong scaffold [34]. Fibrin is another protein that helps stop bleeding by entrapping platelets during the formation of the primary clot [35]. The mechanical strength of silk fibroin also makes it efficient. Keratin is another nanomaterial that sees extensive use in the wound healing process [36]. There is tremendous potential in the fact that nanomaterials can be used to facilitate regenerative self-healing mechanisms. However, due to the diverse character of wounds, it is necessary to learn more about the fundamental processes and cellular cascades in order to tailor those nanomaterials to specific wound healing needs [37].

## 4. Available Therapeutic Approaches in Wound Healing

Biological-based techniques such as immune-based antimicrobial compounds (for example, polypeptides including defensins), therapeutic microorganisms (for example, bacterial phages and probiotics), stem cell treatments, and skin tissue engineering are presently available therapeutic possibilities [38,39]. Reactive species and nitric oxide generators, different topical antiseptics including antibiotics, negative pressure, and ultrasonic treatment, along with manual wound debridement, various surgical techniques, and skin replacements are among the classic and non-traditional non-biological approaches [40]. Owing to variations in the molecular and cellular pathways between wound types, there is not yet a universally effective treatment for wounds [41]. The evaluation and management procedures in the field of wound healing research have been greatly enriched by recent breakthroughs and technological advancements [42]. Changes in the way wounds are treated, such as the introduction of moist dressings, stimuli-responsive dressings, as well as growth factor-based therapy, skin alternative treatments (tissue-engineered and bio-engineered), gene treatment, nanotherapeutics, as well as stem cell therapy, resulted in a new era of progress [43,44,45,46]. Emerging technologies, such as three-dimensional bioprinting (3D bioprinting), novel platelet-rich plasma therapy, including extracellular matrix (ECM)-based techniques, have also paved the way for tailored wound care [47]. However, there is still cause for concern regarding the evaluation and management of chronic wounds. As a result, we need cutting-edge approaches to wound care that take into account efficacy, benefit-to-risk, and cost-effectiveness [48].

## 5. Applications of Different Nanomaterials in Wound Healing

Nanoparticles, with their high surface area-to-volume ratio, are well-suited for use in this type of study. Wound dressings could benefit from the incorporation of metal nanoparticles like silver, gold, and zinc due to their exceptional features, such as wound healing stimulation and antibacterial properties [49]. Rapid wound closure with little scarring has been reported after treatment with silver nanoparticles, which have been shown to influence the release of anti-inflammatory cytokines [50]. They can also promote keratinocyte proliferation, which is essential for epidermal re-epithelization. This last influence appears to be dose-dependent as well. Gold nanoparticles (AuNPs) can boost keratinocyte development and differentiation at low concentrations while also promoting healing and preventing microbial colonization [51]. When incorporated into hydrogel-based wound dressings, zinc oxide nanoparticles (ZnONPs) indicate an efficacious antibacterial agent by triggering bacterial cell membrane perforations, and by increasing the overall contact time, they promote keratinocyte migration and improve re-epithelialization [52]. In addition, the antibacterial and re-epithelialization characteristics of biopolymeric nanoparticles as treatments for wounds or as delivery vectors are impressive [53]. In particular, biopolymer’s high water absorptivity makes it ideal for setting up a humid wound environment and controlling exudation [54]. Hyaluronic acid, by virtue of its hygroscopicity, controls cell adhesion as well as attachment in the course of wound healing. Endothelial cell proliferation, motility, and angiogenesis were all reported to be boosted by hyaluronan oligosaccharides due to elevated levels of vascular endothelial growth factor [55,56].

Infection prevention, skin regeneration, and therapy for wounds are all intricate parts of the healing process. Both topical and systemic treatments are used to aid with wound recovery [57]. However, local therapy may be preferable since it reduces the risk of complications, boosts efficiency, and helps combat antibiotic resistance [58]. Studies have shown that many different pharmacological NPs have remarkable effectiveness in promoting skin regeneration, preventing infections, and inhibiting the growth of multidrug-resistant bacteria [59]. Significant improvements in wound care may result from the synthesis, manufacture, and therapeutic relevance of these NM compounds [60].

By increasing the degree of antibiotic concentration, local antimicrobial therapy helps eliminate wound infections [61]. To reduce the risk of antibiotic-related side effects and resistance, antibiotics should be limited to site applications rather than systemic and long-term therapy [62]. Injuries to the skin’s protective barrier increases the risk that drugs applied topically, including NPs, will be absorbed systemically [63]. Therefore, studies of these NPs as antimicrobials and carriers are essential in order to discover more about their kinetics as well as dynamics, cytotoxicity (eukaryotes), and likely systematic adverse reactions [64]. Furthermore, when employing topical treatments, it is important to consider the effect that cleanup techniques have on the NPs’ half-life within the wound site [65]. Below, we present the many nanomaterials that can be used for wound care. While we do provide a sample of case studies demonstrating the applicability of each type of nanomaterial, we do not publish an exhaustive catalogue of all studies involving any given nanomaterial.

## 6. Carbon-Based Nanomaterial

Deoxyribonucleic Acid (DNA) is the primary vital component of all known forms of life on Earth. It could be made available in a number of different formats. Carbon’s peculiar electron configuration (1s2, 2s2, 2p2) allows for the possibility of both crystalline and amorphous forms of the element. Carbon is a highly versatile element since it may exist in a wide variety of allotropes and crystal structures. The type of carbon allotrope depends on whether the carbon is hybridized (sp3, sp2, or sp1) or bound to other atoms. Carbon-based nanoparticles (CBNs) have garnered interest from several disciplines due to their distinctive physicochemical properties and structural properties [66]. Nanomaterials made of carbon are becoming increasingly popular due to their novel size and promising features, which include mechanical, chemical, thermal, electrical, as well as optical properties. Nanotubes (carbon-based), notably their nanocomposites, have been shown to have broad antibacterial activity [67,68]. Burn wound infections are triggered by bacteria, fungus, and viruses. Knowing the specific mechanisms that govern CNMs’ antibacterial activity is crucial for ensuring their safe and effective inclusion into biomaterials. Carbon-based nanomaterials offer attractive physical and chemical characteristics that could have uses in biomedicine [69,70]. In addition to improved optical activity and a substantial multifunctional surface area, these materials have showed increased drug-loading capacity, greater biocompatibility, and lower immunogenicity. Research into enriched carbon-based nanomaterials has yielded significant benefits for the development of biologically compatible scaffolds and nanomedicines [71,72].

### 6.1. Carbon Nanotubes

The most common form of carbon nanotubes (CNTs) is a tube or cylinder. Size, shape, thickness, and chirality are all possible distinctions between them. They displayed numerous desirable characteristics because of their unusual blend of pliancy, toughness, and rigidity. Single-walled, double-walled, multi-walled, and functionalized CNTs exist because of their varied structural configurations. CNTs can be synthesized using a variety of techniques, including chemical vapor deposition, laser ablation, arc discharge, or, for high-pressure regions, carbon monoxide disproportionate. As Sumiolijima in 1991 mentioned, CNTs constitute an allotropic type of carbon with similar qualities to graphene [73]. CNTs have a cylinder shape and are made up of rolled graphene sheets that are sp2-hybridized. CNTs are often manufactured via arc discharge, laser ablation, or chemical vapor deposition (CVD) [74]. While the carbon source was heated at 3000–4000 °C in the arc discharge as well as during the laser ablation procedures to create cylindrical CNTs, the carbon source was pyrolyzed at 600–1100 °C in the CVD method. The synthetic route taken has a significant impact on the resulting CNTs’ physicochemical qualities [75]. Because of their unique structure and fabrication method, CNTs have exceptional thermal characteristics. Depending on whether they are single-walled or multi-walled, the conductivity of CNTs can range from 6000 to 0.1 W/mK [76,77]. Collective atomic vibration, encompassing phonon and electron transport, is responsible for thermal conductivity [78,79]. The conductivity of CNTs is also dependent on their length [80]. Therefore, in order to achieve a targeted thermal conductivity, it is necessary to optimize synthetic parameters [81]. Based on the Tersoff–Brenner potential, which is analogous to a hypothetical isolated graphene monolayer [82], Berber et al. used molecular dynamics simulation to calculate the thermal conductivity (k = 6600 W/mK) of CNTs. As a counterexample, Osman et al. investigated how CNTs’ physical characteristics affected their heat conductivity. They looked at the fact that SWCNTs’ thermal conductivity varies with temperature. Armchair (10,10) arranged SWCNTs like monolayered graphene and showed a drop in thermal conductivity over 400 K. Maximum conductivity is found in CNTs of equal diameter but differing chirality at 300 K [83,84], with the armchair CNTs having a relatively sharper peak than the zigzag CNTs. A composite film made of bacterial cellulose and multi-walled carbon nanotubes (MWCNTs) was created by Khalid and her colleagues. The antibacterial properties of the finished films were tested. All of the bacteria strains put to the test were successfully inhibited by the composite film. Furthermore, macroscopic inspection of the wound showed that the BC-MWCNT group healed their diabetic wounds more quickly than the negative control group (77% vs. 99% recovery in 21 days). Histological tests corroborated the findings, showing that the BC-MWCNT-treated group had full re-epithelization of the epidermis as well as normal granulation tissue. Studies at the molecular level suggested that the faster healing time in the BC-MWCNT group was likely due to the lower expression of pro-inflammatory cytokines IL-1 and TNF- and the increased expression of VEGF compared to the control [85]. To regulate the gradual release of isoniazid, Chen and his colleagues developed a nanoparticle formulation composed of chitosan and carbon nanotubes. The dimension of chitosan/carbon nanotube nanoparticles has been found to be between 150 and 250 nm using transmission electron microscopy and nanoparticle tracking as well as analysis. The release of isoniazid was greatly extended by chitosan/carbon nanotube nanoparticles, by 48 h. In vitro studies demonstrated that isoniazid cytotoxicity and inflammation could be mitigated by chitosan/carbon nanotube nanoparticles without destroying the drug’s biological function. The wound area was reduced by 94.6% in the isoniazid group and by 89.8% in the isoniazid/carbon nanotubes group. Immunohistochemistry analysis revealed a marked reduction in CD3+ and CD4+ T cell count in the isoniazid/chitosan/carbon nanotubes group [86]. Kittana et al. [87] have synthesized complexes of chitosan with both single- and multi-walled carbon nanotubes (C-SWCNTs and C-MWNTs, respectively). After assessing the produced compounds using TEM, it was found that a structure composed of carbon nanotubes had successfully formed, interlaced within the chitosan polymer. Fibroblast viability and the ability to structure and bind the extracellular matrix were confirmed in tests of synthetic connective tissue. Both forms of complexes were found to enhance re-epithelialization of healed wounds in vivo, but they also resulted in a higher rate of fibrosis in some wounds. These complexes, including the chitosan–multi-wall carbon nanotube composite in particular, were found to increase collage deposition in vitro, correlating with in vivo results that indicated a concentration-dependent increase in fibrosis extent. As a result of these findings, inflammatory symptoms in the bed of the wound increased.

### 6.2. Single-Walled Nanotube

SWCNTs, or single-walled carbon nanotubes, are essentially a long, folded sheet of graphene. Graphene sheet patterns are determined by the diameter as well as the C-C orientation [88]. The length-to-diameter ratio in this case is close to 1000-to-1. They have been built with sp2 hybridization and have one-dimensional hollow as well as cylindrical shapes. These CNTs can grow to lengths of thousands of times their original diameter [89]. They have numerous uses in nanotechnology. To obtain SWCNTs of a high quality and purity, regulated catalytic conditions are needed. SWCNTs have found widespread application as scaffolding for cellular culture growth and as diagnostic devices in the biomedical sector. Due to their strong interaction, SWCNTs have a low solubility and are challenging to distribute in the aqueous phase. Solubilizing and dispersing SWCNTs in water through chemical modification has been proposed to boost its biological and pharmacological interest [90,91].

### 6.3. Double-Walled Nanotube

DWCNTs, or double-walled carbon nanotubes, are originated by joining together two single-walled CNTs. About 2–4 nm is the outer tube diameter, and 1–3 nm is that of the inner tube. DWCNTs possess the same small diameters, mechanical properties, and electrical properties as SWCNTs. Double-walled carbon nanotubes (DWCNTs) have a graphene sheet folded on top of itself twice, creating a double-layer structure. It is a newly discovered type of CBN [92]. DWCNTs excel in these areas compared to SWCNT parameters such as mechanical strength, thermal stability, as well as chemical resistance. The optical and electrical properties of this group are also rather intriguing. As an electrode material, anti-salmonella was injected into DWCNT bundles. DWCNTs, as opposed to the SiO_2_ surface, are preferable for the growth of neuron cells in tissue engineering. After neuron growth on DWCNTs, cell differentiation was enhanced [93].

### 6.4. Multi-Walled Nanotube

The diameter of a multi-wall carbon nanotube (MWCNT) ranges from 2 to 50 nm; the diameter of the outermost layer can be as high as 100 nm, while the diameter of the innermost layer is less than 1 nm. The amount of rolled-up graphene sheets in MWCNTs increases the number of structural flaws that benefit surface functionalization. As a result of their exceptional chemical, thermal, mechanical, electrical, and changeable structural properties and functions, MWCNTs are being evaluated as possible materials for biomedical applications. The Russian-doll and parchment model [94] works well to describe the structure of MWCNTs. When contrasted with DWCNTs and SWCNTs, they are found to have a better signal-to-noise ratio. Sensitivity, however, is conditional on the nature of the matrix (biological). The scaffolds for pancreatic cancer cells have been developed using MWCNTs. The proliferation and growth of fibers/sheets based on validated materials show greater potential in killing cancer cells [95].

## 7. Nanostructured Carriers

Nanomaterials may be made in many different sizes and structures and are generally spherical and have a diameter of 10–100 nm [96]. A wide range of organic compounds and metals can be used to synthesize nanomaterials. Most NPs are made up of two parts: the core compounds or atoms and a surface “cap” or shell that affects the NPs’ stability and also their function [97,98]. The efficacy of NPs is influenced by the interaction of an infectious organism with damaged cells in the wounded region, which is influenced by different biomaterial assets including their nature, dosage form, particle size, as well as the intensity of infectious injury [99]. NPs act as drug carriers that mainly operate as a vehicle to transport medicine to the targeted site more effectively in comparison to the standard topical medicament [100]. Organic (such as surfactant/lipid), carbohydrate-based (e.g., cellulose), or inorganic carrier NPs may be used (e.g., silicate or metal-based). The antimicrobial/drug may be encapsulated in the carrier NP, as in liposome-based nanomaterials, or the antimicrobials may be part of the shell, making the NP the carrier [101]. Surface nanoengineering, which is associated with core–shell nanomaterials, aims to minimize biofilm development in wounds and infections on implantable devices that are medicated [102]. The goal of surface nanoengineering is to develop a coating material that either passively inhibits bacterial growth or actively inhibits bacterial development by permanently attaching antimicrobials to the polymer (non-release-based) or releasing them (either with or without bacterial stimulation) [103,104,105,106].

### 7.1. Liposomal Delivery

**Figure 2:**
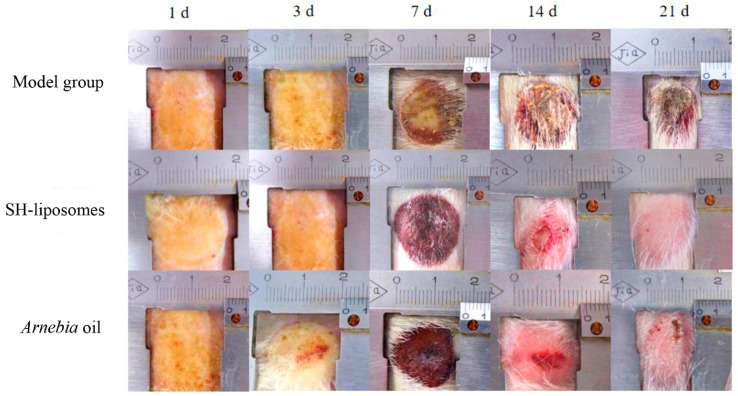
Out of the model group, Shikonin (SH)-based liposomal group, and *Arnebia* oil^®^ group, applied topically twice daily for 21 days, SH-liposomes observed a better wound healing effect on the 21st day compared to the others (reprinted from [116], copyright (2024), with permission from Elsevier).

Liposomal drug delivery helps in the facile transfer of hydrophilic compounds including small molecules as well as non-Lipinski-based macromolecules in varied applications, especially wound healing [107,108,109]. The phospholipid building components are responsible for the amphipathic character of liposomes (LP). The amphiphilic lipid spontaneously forms its bilayer with an aqua base when dispersed in aqueous solutions. The ultimate size of the liposome is determined by the lipid type and extrusion across membranes of varying pore sizes [110,111]. These LPs can also be derivatized with biopolymers to improve their stability and targeting abilities [112]. Towards this direction, Eid et al. in 2022 [113] explored the viable usage of citicoline-tailored chitosan-engulfed liposomes for efficacious topical wound healing, especially in the case of streptozocin-induced diabetic animal models. The study acquired optimized formulations with the average particle size at 211 nm, EE as 50%, and surface area as +32 mV approximately. These liposomes resulted in sustained-release behavior and decreased inflammatory process, thus initiating a VEGF immune reaction, fibroblast development, as well as enhanced epithelialization. Recently, Daodu and coworkers [114] developed curcumin-loaded liposomal delivery inside lysine and collagen-based hydrogel for the improvement of surgical wounds by thin-film hydration process. These infused dosage forms were of 5–10 microns in particle size, EE was 99.9% approximately, and they exhibited 79% of increased wound contraction after the third day of operation. Hemmingsen et al. in 2022 [115] designed another chitosan-based chlorhexidine delivery system for antimicrobial action. The inflammatory pathways in treated rodent macrophages showed a 60% reduction in comparison to the non-treated animals. The novel formulation has indicated an excellent antimicrobial action against *Staphylococcus aureus*. Shu and coworkers in 2022 [116] fabricated a herbal antimicrobial wound healing preparation bearing shikonin-based LP using a film development process against methicillin-resistant *S. aureus* (MRSA). The LPs were stable, exhibited an EE of 62%, were negatively charged, and had a sustained-release effect. The preparations damaged the bacterial cell membrane, alleviated the inflammatory pathway, and showed better repair activity (Figure 2).

Another group investigated chitosan-coated liposomal compositions for delivering substance P (SP), an 11-amino-acid neuropeptide that aids in mucosal wound healing. Liposomes were produced using lecithin and cholesterol, then loaded with SP and resuspended in water before being coated with chitosan. The diameter of the uncoated liposomes (UN-LP) was 151 ± 27 nm, whereas the diameter of the coated liposomes (CH-LP) was 243–248 nm. The SP-infused chitosan-functionalized liposomes were tested in vitro against HaCaT keratinocyte cells to see if they could repair wounds. When cell migration was monitored, it was shown that SP incorporated in chitosan-coated liposomes led in wound repairs and closure was of 85.512%, which was much greater than untreated cells [117]. Notably, the plant origin extract curcumin has revealed wound healing capabilities. Deformable liposomes (DL) in chitosan hydrogel were used for its therapeutic action. Both DLs and chitosan hydrogel assured the drug’s long-term release and penetration. A longer retention duration at the targeted skin location, better localized wound treatment, and increased medication effectiveness were its positives [118].

### 7.2. Nanohydrogels

**Figure 3:**
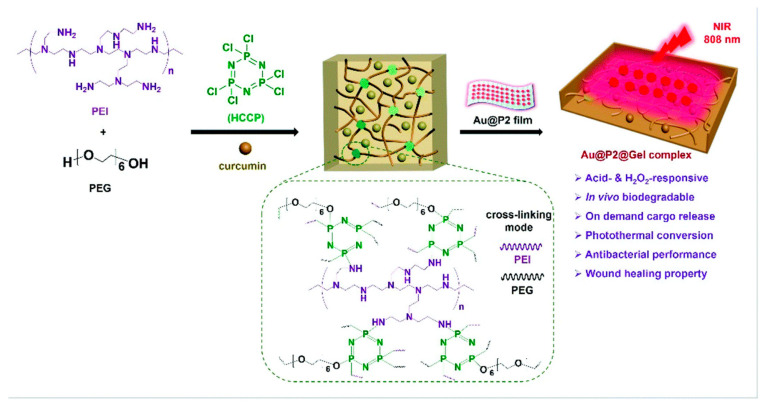
Schematic representation of hydrogen peroxide (H_2_O_2_)-responsive nanohydrogel with a photothermal conversion ability based on polyethylenimine (PEI), polyethylene glycol (PEG), hexachlorocyclic triphosphonitrile (HCCP), as well as gold nanoparticles. Reproduced from [125] with permission from the Royal Society of Chemistry.

Interestingly, hydrogels have proved to have an advanced efficacy with the incorporation of nanomaterials and have made wound dressings more pragmatic based on research studies. Merging nanotechnology with hydrogels has shown improvements in the adhesive properties of these gels and the potency of drug delivery through covalent coupling and noncovalent interactions. Henceforth, nanohydrogels can be employed directly on the wounded site and can cover the damaged area to enhance wound healing as well as the growth of hair follicles and capillaries [119]. Many research studies have observed that different functionalities bearing hydrogels have enhanced pharmacological healing activity in comparison to the standard formulations. The modern advantages of hydrogel-based dressings are their antibacterial, surface adhesion, hemostasis, anti-inflammatory, antioxidant, target delivery, self-healing, stimuli-responsive, and wound diagnostic features [120]. Bilayer hydrogels have shown tremendous applicability, especially in wound healing action [121,122]. In fact, a few compounds, such as hyaluronic acid, have demonstrated excellent flexibility in the pharmaceutical industry owing to their gel-based nature [123]. Furthermore, Sun et al. in 2022 [124] discovered that a novel oxidized konjac glucomannan (OKGM), γ-poly(glutamic acid), could be modified with dopamine and L-cysteine (γ-PGA-DA-Cys) and ε-polylysine (ε-PL) to produce an OKGM/γ-PGA-DA-Cys/ε-PL (OKPP) bioadhesive hydrogel. The formulation was active against *P. aeruginosa* and *S. aureus*, showing better antibacterial as well as antioxidant activity. The animal model observed efficacious wound repair properties and decreased inflammatory parameters, leading to a potential wound dressing for burn infections. To our knowledge, Shang et al. in 2022 [125] demonstrated a versatile nanohydrogel-based wound dressing that observed biodegradability and multiple biological effects including antimicrobial, antioxidant, and healing action against MRSA. This construct was an acid-cleavable antibacterial formulation bearing a hydrogen peroxide (H_2_O_2_)-responsive polymer/gold hybrid film with a photothermal conversion ability based on polyethylenimine (PEI), polyethylene glycol (PEG), hexachlorocyclictriphosphonitrile (HCCP), as well as gold nanoparticles. These gels showed notable surface adhesion and an easy-to-remove feature, which may lead to patient compliance (Figure 3).

### 7.3. Metals and Metal Nanoparticles as Antimicrobials

Bacterial infectious illnesses are a major public health concern because they indicate a global pandemic. Around the world, resistant diseases kill at least 700,000 people per year. Since antibiotics are so powerful against these illnesses, they have become the standard for both prevention and treatment [126]. However, numerous studies have found that antibiotics currently in widespread use are the leading sources of life-threatening multidrug-resistant bacteria. Between 2017 and 2019, the FDA authorized eight novel antibiotics developed to combat WHO priority infections. Many of these medicines are variants of traditional antibiotic classes already in use. Nanotechnology, defined as nanoscale materials (1–100 nm), is a promising field in modern biomedical applications. These materials can improve physicochemical and biological qualities because of their unique properties [127,128,129].

Metal-based nanoparticles (MBNPs) are being examined extensively for a variety of biological applications, and they provide hope in the fight against antibiotic resistance. Additionally, they target several biomolecules, which further hinders the evolution of resistant strains of bacteria, and their mechanisms of action are completely distinct from those outlined for conventional antibiotics [130]. Nanomaterials have been shown to be hazardous to multiple bacterial strains in in vitro tests, raising hopes that they may one day be used in medicine delivery as well as tissue engineering [131].

Antibacterial wound dressings, including a variety of intermetallic NPs and polymeric biomaterials, have been reported in a number of investigations. In this direction, researchers have developed antibacterial cellulose-PMMA fibers with a pressurized gyration process for epidermal wound dressing applications. Dressings with the highest NP concentrations (1 wt.%) for both blends were tested by in vitro co-culturing *S. aureus* and keratinocytes to determine the cellular response and the concomitant bactericidal effectiveness. Compared to samples containing UHNP-1 intermetallic NPs, samples containing AVNP-2 intermetallic NPs demonstrated superior antibacterial activity and keratinocyte cell survival. The fibers showed great potential as a wound treatment for the epidermis. The aforementioned research suggests that wound dressings composed of intermetallic NPs–polymeric biomaterial systems can efficiently kill many bacterial strains [132,133,134].

Antibacterial wound dressings based on intermetallic NPs–antibiotic/polymeric biomaterial complexes are currently poorly understood. Using intermetallic NPs to decrease antibiotic dosage and polymeric biomaterials to increase their sustained bactericidal activity would be an exciting field of study [135,136]. One of the most prevalent ways in which MBNPs inhibit the growth of bacteria is by inducing oxidative stress through the release of reactive oxygen species (ROS). Overproduction of reactive oxygen species (ROS) causes cell death in bacteria by damaging their membranes, DNA, ribosomes, and proteins, and by inhibiting their electron transport chain, enzymes, and DNA transcription and translation [137,138]. It has been established that most MBNPs can enter cells and trigger the production of reactive oxygen species (ROS), while ROS production outside of cells has also been recorded. The microbial membrane experienced elevated oxidative stress because free electrons from Ag NPs that were embedded in Ti reacted directly with O_2_ in the liquid culture medium. The levels of reactive oxygen species (ROS) in bacteria can be increased by NPs, but bacteria have a defense mechanism termed hormesis that can respond to ROS [139,140].

Hormesis is a short- and long-term defense mechanism against reactive oxygen species (ROS), with the former being activated by a sudden spike in ROS concentration and leading to the development of ROS scavenger enzymes, and the latter being increased at the transcriptional level. So, hormesis should be taken into account while designing MBNPs–antibiotic/polymeric biomaterial systems. In addition, MBNPs can bind to the cell membrane and cause damage, which can lead to the release of intracellular material or the clogging of transport channels. Furthermore, MBNPs can dissociate into ions in solution that can cross the cell membrane. The production of proteins and DNA can be hampered by ions because of their ability to bind to functional groups. Many different modes of action have been proposed, and it is believed that several of these processes can operate in combination [141,142,143,144].

### 7.4. Silver Nanoparticles

Healing a wound requires a series of coordinated events, including clotting, inflammation, tissue deposition, fibroplasia, differentiation of the extracellular matrix, cellular contraction, tissue remodeling, and, finally, epithelialization. In order to combat bacterial growth in long-standing wounds and burns, doctors frequently turn to silver compounds like silver nitrate as well as silver sulfadiazine [145,146]. The faster healing of wounds caused by diabetes is achieved with the use of AgNPs, which promote the differentiation of fibroblasts into myofibroblasts. By promoting keratinocyte proliferation and migration, AgNPs hasten the healing process. It is possible that AgNPs will interact with sulfate-containing proteins in bacteria membrane cells, leading to an attack on the respiratory chain and, ultimately, apoptosis. Neutrophil apoptosis is triggered upon contact with the wounded area because mitochondrial function is reduced, which in turn decreases cytokine production [147,148]. This results in a less inflammatory response, which in turn promotes quicker recovery. However, AgNPs are able to cause DNA damage, suppress cell proliferation, and stifle cellular ATP generation because of their small size, which allows them to easily infiltrate biofilms and cell membranes. The presence of silver nanoparticles in a wound alters the local concentration of messenger RNA. Collagen deposition, which is associated with the migration of macrophages and fibroblasts, increases as the concentration of AgNPs in the dressings rises. Decreased silver ion toxicity and increased local antibacterial activity can be achieved through the use of sustained-release mechanisms [149,150,151,152].

Using a box behnken design, Salem et al. 2022 [153] developed and optimized RVS-loaded nanocubics encapsulated with AgNPs to test their efficacy in wound treatment. Various pharmacological studies were conducted on rats to determine the efficacy of wound healing when the optimal formulation topped with AgNPs was added to a gel foundation. The combination of RVS-loaded nanocubic vesicles as well as AgNPs-loaded hydrogel has shown promise as a platform for improving wound healing and tissue restoration. Wound dressings with silver nanoparticles and antibiotics (ciprofloxacin) were developed by Massey et al. in 2022 [154]. Cross-linking the hemicellulose from Lallemantiaroyleana seeds with chitosan/chitin and glutaraldehyde improved the hemicellulose’s absorptive capabilities. It was determined that the dressings were permeable and that the silver nanoparticles as well as the drug particles were dispersed evenly throughout the polymeric matrix. Because of its high swelling index, porosity, and spongy texture, it has been found useful in the creation of wound dressings with enhanced characteristics.

### 7.5. Zinc Oxide Nanoparticles

A number of beneficial effects of zinc oxide nanoparticles (ZnO NPs) have been reported. Cell migration, re-epithelialization, as well as angiogenesis are all boosted by ZnO NPs. These characteristics are crucial for the recovery process after an injury. The extraordinary antibacterial capabilities of ZnO-NPs have contributed to their development as potent agents against MDR. ZnO-NPs also exhibit outstanding photo-oxidation and photocatalytic features [155,156]. It is widely believed that ZnO-NPs’ antibacterial efficiency can be attributed to oxidative stress, DNA damage, and photocatalytic activity, even though the mechanism of ZnO-NPs’ antimicrobial action is not fully established. Oxygen annealing of ZnO enhances the amount of oxygen atoms on its surface, which increases oxygen atom adsorption as well as leads to the formation of more ROS, which boosts the efficiency of oxidation and, by extension, facilitates the material’s antibacterial property, as reported by Sirelkhatim et al. It has been discovered that ZnO-NPs directly interact there with the cell membrane of bacteria, disrupting its integrity, making them a potent bactericidal agent versus both Gram-positive and Gram-negative bacteria [157].

### 7.6. Titanium Oxide Nanoparticles

TiO_2_ has antibacterial characteristics, making it a promising biomaterial for use in wound treatment. Because of its high biocompatibility, TiO_2_ is used in a wide variety of medicinal fields [158]. Additionally, there is probably a low danger of bacterial resistance acquisition with the clinical usage of TiO_2_ in dressings for wounds. Soft silicone and alginate were used as hydrophobic and hydrophilic dressing materials, respectively, and TiO_2_ was deposited on them, employing the atomic layer deposition technique. Flexible dressings were maintained without sacrificing durability thanks to a nanoscale TiO_2_ covering [159].

In 2022, Aleem et al. [160] cross-linked chitosan and cellulose with sulfur-doped titanium oxide nanoparticles using triethyl orthoformate. In comparison to the other tested materials, membranes loaded with titanium oxide nanoparticles (CLHTS-5 wt%) showed the highest level of angiogenesis. Furthermore, it was concluded that a decrease in lactate dehydrogenase enzyme release was observed for up to 72 h after the wound developed, highlighting the promising ability of these nanoparticles in applications related to wound healing. Ismail et al. 2021 [161] developed a biofilm wound dressing using an evaporative casting method that included titanium dioxide nanoparticles integrated in gellan gum (GG + TiO_2_-NPs). Within 14 days, the GG + TiO_2_-NPs biofilm-treated wound was completely healed, and there were no harmful effects on the mouse fibroblast cells. Titanium nanoparticles (TiNPs@Ziziphora) were developed by Mahdavi et al. (2019) [162], utilizing an aqueous extract of Ziziphoraclinopodioides Lam leaves and TiO_2_ nanoparticles at Ziziphuschandleri. The antibacterial and antifungal activities of synthesized nanoparticles were shown to be superior to those of all currently available conventional antibiotics. When compared to the control groups, those given the TiNPs@Ziziphora ointment saw significant improvements in wound closure, hydroxyl proline, hexosamine, hexuronic acid, and fibrocytes/fibroblast rate, and a decrease in wound area, total cells, neutrophils, and lymphocytes (all *p* ≤ 0.01).

### 7.7. Gold Nanoparticles

Due to their high absorption of near-infrared (NIR) light, facile manufacturing, and chemical stability, gold nanoparticles (AuNPs) have found applications in tissue restoration, wound healing, and intelligent drug delivery. To aid in the in vivo transcutaneous co-delivery of other medications, gold nanoparticles traversed the subcutaneous layer of skin and disseminated across the epidermis [163]. When applied to open wounds in Wistar Albino rats, AUNPs boosted collagen as well as granulation tissue deposition, accelerated re-epithelialization, and hastened wound healing. Adenosine triphosphate (ATP) is the main molecule for storing and transmitting energy in cells, and gold nanoparticles stopped the activity of *P. aeruginosa* as well as *S. aureus* by inhibiting their energy metabolism and the activity of the enzyme that produces ATP, adenosine triphosphate synthase [164]. The biocompatibility, biodegradability, and free radical scavenging activity of AuNPs are enhanced by bridging between collagen, gelatin, and chitosan. When compared to chitosan alone, the chitosan–AuNPs composite enhanced hemostasis and epithelial tissue development in injured rats. Cryopreserved human fibroblasts, which were treated with AuNPs, were applied topically to burn sites, where they expedited healing by decreasing inflammation and increasing collagen deposition [165,166].

**Figure 4:**
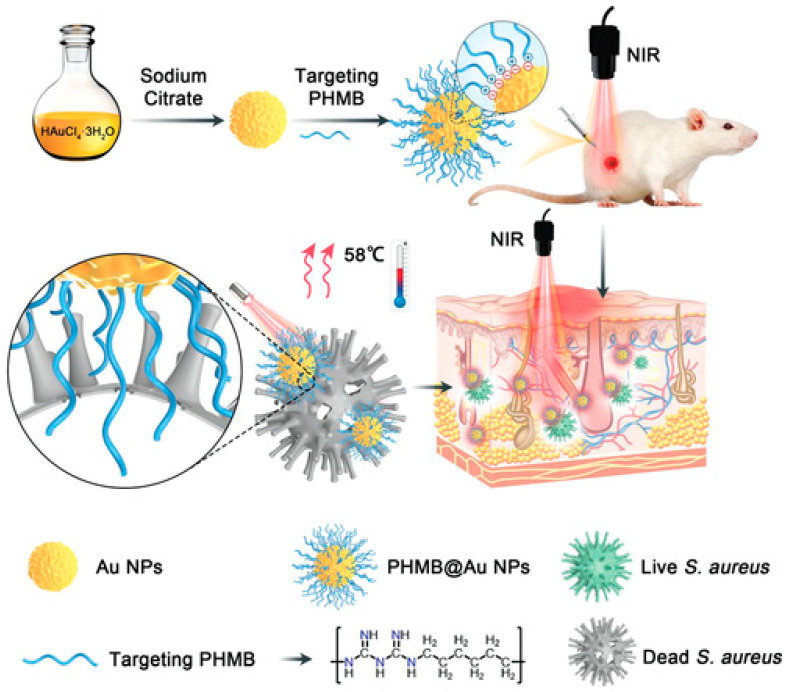
Diagram depicting the production of Au NPs and PHMB@Au NPs, as well as an analysis of the mechanism of their bactericidal impact in vivo and their ability to expedite the healing of infected wounds [167]. (https://doi.org/10.1002%2Fadvs.202105223 is licensed under https://creativecommons.org/licenses/by/4.0./, accessed on 15 November 2023).

Polyhexamethylene biguanide–gold nanoparticle (PHMB@Au NPs) hybrids were developed as an antibacterial agent by He et al. in 2022 [167]. This platform is a promising antibacterial agent in wound healing since its synergistic action greatly improves the photothermal bactericidal activity against *Staphylococcus aureus* when exposed to near-infrared irradiation (Figure 4).

Hydrogels based on gold nanoparticles (Au NPs) were synthesized by Batool et al. [168] from Cydonia oblonga seed powder, which has been shown to have improved antibacterial and wound healing properties. In vivo treatment of wounds with hydrogel-based Au NPs indicated that Au NPs in murine models demonstrated a 99% closure of the wound within 5 days, and the hydrogel-based Au NPs suppressed microbial development in zones with a diameter of 12 mm.

### 7.8. Nanofibers

In addition to the utilization of NP, nanofibers are promising for wound healing due to their unique advantages. Their high surface area facilitates enhanced cell attachment, proliferation, and migration, fostering expedited wound closure and tissue regeneration. Additionally, their high porosity ensures breathability, prevents fluid accumulation, and inhibits bacterial growth, fostering a moist wound healing environment crucial for optimal recovery. Nanofibers, crafted from biocompatible materials like collagen and chitosan, mitigate the risk of allergic reactions, ensuring safe application on the skin. Moreover, their ability to carry drugs and bioactive molecules enables targeted delivery, enhancing treatment efficacy [169,170].

**Figure 5:**
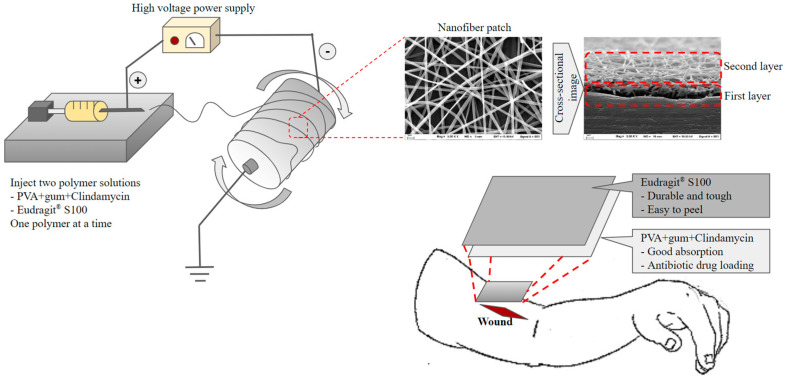
Preparation and application of the bilayer wound healing patch nanofiber fabricated by electrospinning.

Kampanart and colleagues [106] have successfully engineered a bilayer nanofiber wound dressing patch using the electrospinning technique (Figure 5). The initial layer included modified tamarind seed gum encapsulating PVA and clindamycin HCl, whereas the subsequent layer consisted of Eudragit^®^ S100. The results obtained from the scanning electron microscopy (SEM) analysis revealed the presence of nanofibers, exhibiting a wide range of diameters, spanning from 153 to 1830 nm. These variations in diameter were observed to be impacted by sprayed solution formulation and equipment setting. The patch was effectively created by utilizing optimum electrospinning conditions. The drug analysis results have proven the presence of an amorphous state. The biological experiments conducted demonstrated effectiveness against *Staphylococcus aureus*. The utilization of Eudragit^®^ S100 and modified tamarind seed gum exhibits promising prospects in augmenting the strength of nanofiber patches.

## 8. Metal Nanoparticles with Biomaterials in Wound Healing

Biomaterials made from metals have been shown to play an active role in wound healing and infection prevention. Research into metal-based biomaterials is an exciting new frontier since they show promise in a wide range of applications beyond simple wound hemostasis, including management of inflammation, encouragement of angiogenesis and re-epithelialization, antibacterial activity, and even multistage combination therapy. Accordingly, we categorize metal-based biomaterials into 0D, 1D, 2D, and 3D categories and review the most recent findings about their use in wound repair based on these dimensions [171].

### 8.1. Zero-Dimensional Metal-Based Biomaterial Platforms in Wound Repair

In the field of biomaterials based on metals, OD materials are those whose dimensions vary only on the nanoscale scale. The biomedical profession makes considerable use of common OD metal-based biomaterials, including angstrom-scale materials as well as metal quantum dots, because of their high biological or photocatalytic activity. OD metal-based biomaterials have potent antibacterial effects during wound repair because of their elevated electron mobility and unique specific surface area. Compared to conventional antibacterial drugs, the therapeutic effects of OD metal-based biomaterials are significantly higher [172]. By penetrating the bacterial membrane and making close contact with the cell, materials with ultrafine structures can reduce bioburden and accomplish their goal of lysing bacteria. Nanosilver has gained a lot of attention as a potentially effective broad-spectrum antibacterial material for treating wounds. To this end, Chen et al. [173] employed a safe as well as rapid physical method to synthesis angstrom-scale Ag nanoparticles (AgPs) and then utilized them with carbomer gel (L-AgPs-gel) to assess their broad-spectrum antibacterial impact on wounds. AgPs cause bacteria to produce more reactive oxygen species (ROS) and hence perish. The infection and inflammation induced by bacterial colonization in the wound are greatly reduced because the L-AgP gel has a higher suppressive inhibitory impact on the biofilm development of numerous pathogens than the readily accessible AgNP gel.

### 8.2. One-Dimensional Metal-Based Biomaterial Platforms in Wound Repair

The term “1D biomaterials” is employed to describe substances that only have a single dimension of extension, with the other two dimensions being on the nanoscale scale. In contrast to OD biomaterials, its strong specific surface area, exceptional photothermal action, catalytic properties, stability, and biological activity render it a suitable material for directing electron transport. Wound repair is a major use case for 1D biomaterials like metal nanorods and nanowires. One-dimensional (1D) biomaterials based on metals are a novel material for facilitating wound repair and angiogenesis [174]. The human body’s tissues rely on angiogenesis to bring in oxygen and nutrients. Biomaterials based on lanthanide metals are increasingly used in the medical industry because of their superior biological and fluorescent properties and because their proangiogenic qualities have been demonstrated experimentally [175,176]. A blend of polycaprolactone (PCL) and TiO_2_ nanorods (TNRs), electrospun nanocomposite mesh was developed by Augustine et al. [177] for use as a wound dressing. The composite material net mimics the ECM and promotes cell adhesion and proliferation, creating an optimal setting for wound healing. TNRs in the PCL grid have been shown to enhance cell migration, proliferation, and angiogenesis in wound healing in both in vivo and in vitro evaluation.

### 8.3. Two-Dimensional Metal-Based Biomaterial Platforms in Wound Repair

High-efficiency biocatalytic activity is provided by two-dimensional metal-based biomaterials, which are characterized by their two-dimensionality (the other dimension having been on the nanometer scale) and their ability to allow molecules of a substrate to interact onto active sites on the outermost layer with a minimal energy barrier [178]. Recent years have seen an uptick in studies examining their potential in the fields of tissue engineering and wound healing. The exceptional mechanical properties of 2D metal-based biomaterials such as nanocoatings/films, 2D transition metal dihalides (2D TMDs), and 2D metal–organic frameworks (2D MOFs) have made them desirable for use in wound healing. When it comes to treating wounds, 2D metal-based biomaterials have shown to be highly successful as novel antibacterial agents, penetrating biofilms and preventing infection [179,180].

### 8.4. Three-Dimensional Metal-Based Biomaterial Platforms in Wound Repair

Three-dimensional biomaterials are composites made up of one or more low-dimensional biomaterial’s basic structural elements [181]. Metal-based biomaterials in the third dimension (compared to other dimensions) typically combine metal-based compounds with non-metallic materials [182]. Because of their complexity, they are more versatile and biocompatible. The regulatory effect of metal elements on wound healing has been mentioned previously, and in recent years, 3D metal-based composite biomaterials such as bioactive glass (BG), mesoporous nanoparticles (MNs), and metal–organic frameworks (MOFs) have been demonstrated to play essential functions in wound management by metal ions and to exhibit better biocompatibility [183,184]. Hu et al. [185] developed copper-doped borate BG/poly (lactic-co-glycolic acid) dressings infused with vitamin E (VE) to stimulate blood vessel formation and skin cell renewal. Enhanced wound angiogenesis, collagen remodeling, and re-epithelialization were observed in studies using the dressing, suggesting its potential utility in wound repair. Feng et al. [186] designed a multilayer composite dressing loaded with BG to promote angiogenesis, with a Janus membrane, which comprises a microporous array for biologically active ions active pumping, an absorbent with effective wound exudate absorption capacity, and a bioactive layer containing BG to stimulate tissue repair and new blood vessel formation.

## 9. Metal Nanoparticle Potential Cytotoxicity

Unlike common metals (such as Li and Na), metal-based NPs display toxicities and biochemical activity that are distinct from those of these elements. Because metal-based NPs are about the same size as protein molecules, they can easily cross cell membranes and accumulate in subcellular organelle structures, where they can trigger a cascade of harmful effects including neurotoxicity, immunotoxicity, and genotoxicity [187]. Numerous studies have demonstrated that many factors, including surface area, particle size, crystal conformity, exposure manner, and chemical components, contribute to the cytotoxicity generated by metal-based NPs. Extensive and well-designed experiments are thus necessary to provide a convincing explanation for the cytotoxic properties of metal-based NPs [188]. Furthermore, the consequences of each parameter must be independently validated when working with metal-based NPs. In order to assess the cytotoxicity of metal-based NPs in a thorough and accurate manner, it is necessary to build a structure–activity correlation model linking the attributes of metal-based NPs and compounds in the body. In order to evaluate the connections between various physical and chemical factors and cytotoxicity, for instance, high-throughput screening technology has been developed. Nanoparticle (NP) and nanobiological system (NBS) interactions can be understood and predicted with the help of structure-based computational molecular modeling [189].

### 9.1. Neurotoxic Effects

Metal-based NPs cause neurotoxicity, which manifests primarily in changes to neurobehavior, histopathology, and neurotransmitters. Changes in neurobehavioral functioning are frequently utilized as a proxy for neurotoxicity caused by metal-based NPs. For example, Ag NPs impaired the social behavior, memory retention, and motor coordination of mice administered with Ag NPs [190]. Cognitive dysfunction and pathological abnormalities in the hippocampus (a brain area directly connected with learning and memory) were demonstrated by Tian et al. In this instance, the cognitive deficits seen in elderly mice were more severe than those seen in adult animals. Despite their widespread application as MRI contrast agents and theragnostic drug transporters, SPIONs may also cause neurotoxicity in the dorsal striatum, including the hippocampus. Metal-based NPs can cause neurotoxicity in children as well as adults. Neurotoxicity has been reported to be a concern for infants and adolescents exposed to metal-based NPs. Children born to moms who have been exposed to NPs made from metals also run the risk of experiencing neurotoxic consequences [191].

### 9.2. Immunotoxicity

The immune system is highly attuned to the presence of foreign chemicals and capable of rapidly mounting a defense against them. Immunity encompasses a wide variety of interactions between innate and adaptive responses, which vary in speed and specificity. Nanoparticles made of metals are more probable to enter organisms as well as interact with one another than other materials. Metal-based NPs, once inside an organism, would have an effect and be removed by the immune system in a way that is determined by their physical and chemical properties [192,193].

### 9.3. Genotoxicity

Substances with deleterious impacts on gene material (DNA and RNA) and on cell integrity are deemed to be genotoxic. Cancer research and the risk assessment of possible carcinogens rely heavily on the study of genotoxicity. It is common to confuse mutagenicity and genotoxicity. Even though genotoxic compounds can cause mutations, not all mutagens do so. This is because not all mutagens cause permanent changes in the genetic material. While a mutagen might temporarily damage DNA or RNA, these damages can sometimes be repaired by cellular mechanisms without leaving a lasting alteration. In contrast, genotoxic substances may also induce other forms of harm that do not necessarily directly alter the sequence of nucleotides, such as chromosomal breaks, rearrangements, or epigenetic modifications. These effects, even if not directly mutational, can still disrupt normal cellular processes and potentially lead to cancerous development. Genotoxicity is caused by metal-based NPs because of their nanoscale size, which allows them to penetrate the nucleus and interact with the arrangement and functioning of genomic DNA. Unfortunately, the precise cause of this genotoxic impact is still unknown. The direct effects of NPs on genomic materials, including ROS production and DNA/chromosome damage, are currently the focus of much study [194,195]. A study discovered that the presence of gold nanoparticles measuring 10 nm altered these markers significantly in rat organs, indicating that they interfere with the antioxidant defense mechanism. The observed rise in free radical concentrations and decline in antioxidant activity may be ascribed to the nanoparticles’ generation of reactive oxygen species (ROS), which may result in detrimental consequences [196]. However, gold nanoparticles with a size of 20 nm, at a concentration of 2.5 mg/mL for 21 days, showed potential therapeutic benefits without toxicity [197].

## 10. Future Challenges

Nanomaterials have emerged as promising candidates for wound healing applications due to their unique properties and potential therapeutic benefits, especially biopolymer nanoformulation. These nanoformulations, composed of biocompatible and biodegradable polymers, offer controlled drug delivery, enhanced wound closure, and reduced scarring. However, as we look ahead to the future, several challenges need to be addressed to maximize the efficacy and clinical translation of these biopolymeric nanoformulations. One of the primary challenges is the optimization of drug release kinetics. Biopolymeric nanoformulations can encapsulate a wide range of therapeutic agents, including growth factors, antimicrobials, and anti-inflammatory drugs. However, achieving the desired release profile of these drugs is critical for effective wound healing. Future research should focus on developing innovative strategies to control the release kinetics, such as stimuli-responsive systems that can respond to the wound microenvironment or external triggers. Additionally, understanding the pharmacokinetics and pharmacodynamics of these nanoformulations in vivo is essential for determining the optimal dosage and frequency of administration. Another significant challenge is improving the stability and shelf-life of biopolymeric nanoformulations. Many biopolymers are prone to degradation and can undergo changes in their physicochemical properties over time, leading to a loss of drug encapsulation efficiency and compromised therapeutic efficacy. Future efforts should concentrate on developing robust formulation techniques and storage conditions that can ensure the long-term stability of these nanoformulations without compromising their structural integrity or drug release properties. This includes exploring novel cross-linking methods, lyophilization techniques, and protective coatings to enhance stability and extend shelf-life. Furthermore, the scalability and manufacturing of biopolymeric nanoformulations remain a challenge. The transition from laboratory-scale synthesis to large-scale production is often hindered by the complexity and variability of the manufacturing processes. Future research should focus on developing scalable and reproducible manufacturing techniques that can ensure the consistent production of high-quality nanoformulations. This may involve the optimization of formulation parameters, the development of continuous manufacturing processes, and the integration of process analytical technologies for real-time monitoring and control. In addition to technical challenges, regulatory and safety considerations are crucial for the clinical translation of biopolymeric nanoformulations. The regulatory landscape for nanomedicine is rapidly evolving, and it is essential to address concerns related to biocompatibility, toxicity, immunogenicity, and long-term safety. Rigorous preclinical and clinical studies are necessary to evaluate the biocompatibility and safety profiles of these nanoformulations. Furthermore, standardization of characterization techniques and guidelines for evaluating the quality, safety, and efficacy of biopolymeric nanoformulations are needed to facilitate regulatory approval and clinical translation. Lastly, the cost-effectiveness of biopolymeric nanoformulations needs to be addressed. While the potential therapeutic benefits of these nanoformulations are significant, their high production costs can limit their widespread adoption and accessibility. Future research should focus on developing cost-effective manufacturing processes, optimizing material selection, and exploring alternative strategies, such as 3D printing, to reduce production costs and enhance affordability.

## 11. Conclusions

The use of nanoparticles in the treatment of wounds has been on the rise in recent years. The most recent advances in nanomaterials that aid wound healing as well as the underlying mechanisms have been covered here. The majority of the published works focus on improving the hemostasis, infection, and immunoregulation, including proliferation; however, there is a knowledge gap about the correct processes and post-wound adjustments. Due to their unique combination of physicochemical and biological features, NPs have shown promise as a means of sustained therapeutic drug delivery and release in wound dressings. Wound healing NPs can also absorb light and convert it to heat or reactive oxygen species, killing any bacteria that may be present in the wound. To treat microbial infections, NPs may be combined with other endogenous triggers including pH level, temperature, enzymes, and toxins released by the bacteria to create an effective wound dressing. Although there has been significant progress in the use of NP-based wound dressings for bacterial detection and treatment, many obstacles, including reliability, stability, toxicity, and histocompatibility, remain in the manner of NPs rendering the transition from the laboratory to clinical. Furthermore, animal trials are typically used to investigate the behavior of NP-based wound dressings in vivo. Since there are significant differences between human and animal models, it is critical to locate a different approach for preclinical investigations. More and more people are interested in and making progress towards developing intelligent wound dressings that can identify and cure bacterial infections without detaching the protective covering from the wound. In this way, bacterial infections can be immediately monitored and treated using the multimodal approach, which provides synergistic and successful therapy. Therefore, NP-based wound healing offers promise for the future diagnosis and treatment of wound infections.

## Data Availability

Not applicable.
